# TP-GCL: graph contrastive learning from the tensor perspective

**DOI:** 10.3389/fnbot.2024.1381084

**Published:** 2024-05-21

**Authors:** Mingyuan Li, Lei Meng, Zhonglin Ye, Yanglin Yang, Shujuan Cao, Yuzhi Xiao, Haixing Zhao

**Affiliations:** ^1^College of Computer, Qinghai Normal University, Xining, China; ^2^The State Key Laboratory of Tibetan Intelligent Information Processing and Application, Xining, China

**Keywords:** graph neural network, graph contrastive learning, complex structure, hypergraph, high-order adjacency tensor

## Abstract

Graph Neural Networks (GNNs) have demonstrated significant potential as powerful tools for handling graph data in various fields. However, traditional GNNs often encounter limitations in information capture and generalization when dealing with complex and high-order graph structures. Concurrently, the sparse labeling phenomenon in graph data poses challenges in practical applications. To address these issues, we propose a novel graph contrastive learning method, TP-GCL, based on a tensor perspective. The objective is to overcome the limitations of traditional GNNs in modeling complex structures and addressing the issue of sparse labels. Firstly, we transform ordinary graphs into hypergraphs through clique expansion and employ high-order adjacency tensors to represent hypergraphs, aiming to comprehensively capture their complex structural information. Secondly, we introduce a contrastive learning framework, using the original graph as the anchor, to further explore the differences and similarities between the anchor graph and the tensorized hypergraph. This process effectively extracts crucial structural features from graph data. Experimental results demonstrate that TP-GCL achieves significant performance improvements compared to baseline methods across multiple public datasets, particularly showcasing enhanced generalization capabilities and effectiveness in handling complex graph structures and sparse labeled data.

## 1 Introduction

In recent years, GNNs have emerged as a powerful tool in deep learning, finding widespread applications in the processing and analysis of complex graph-structured data. GNNs have demonstrated significant potential in various domains such as social network analysis (Min et al., 2021; Kumar et al., 2022; Wei et al., 2023; Xu et al., 2023), recommendation systems (Gao et al., 2023; Liu S. et al., 2023; Sheng et al., 2023), and bioinformatics (Wang et al., 2023; Zhao et al., 2023). In contrast to traditional neural networks, GNNs are characterized by their unique message-passing approach, allowing iterative propagation and aggregation of information between nodes, thus enriching and enhancing the representation of nodes. Across multiple downstream tasks, including node classification (Shi et al., 2021; Lin et al., 2023; Zou et al., 2023), link prediction (Liu et al., 2022; Liu X. et al., 2023), and graph classification (Zhou et al., 2023), GNNs have exhibited outstanding performance.

However, despite the impressive performance of GNNs in semi-supervised learning, their dependence on labels also imposes certain limitations on their applicability to unlabeled datasets. In semi-supervised learning, the challenge of insufficient labeled data becomes increasingly prominent, and GNNs typically require a substantial amount of label information to guide the model in learning accurate node representations (Kipf and Welling, 2016; Hamilton et al., 2017; Veličković et al., 2017). This limitation may become more significant in practical applications, particularly when dealing with large-scale graph data or domain-specific tasks.

To overcome this challenge, researchers have turned their attention to self-supervised learning as a potential solution (Wu et al., 2021; Liu et al., 2022). Self-supervised learning not only addresses the issue of sparse labels but also explores the inherent latent structures and features within graph data, leading to more generalizable representations (Kim et al., 2022). Graph contrastive learning, as a strategy within self-supervised learning, revolves around the core idea of driving graph representation learning by understanding the similarity between different parts of a graph (Zhu et al., 2021; Shuai et al., 2022). This method does not rely on external label information but instead leverages the internal structure and features of the graph to design contrastive tasks, aiming to maximize the exploration of latent information within the graph data. By comparing different nodes and subgraphs within the graph, graph contrastive learning guides the model to learn more abstract and generalizable representations. Particularly in the context of semi-supervised learning, graph contrastive learning methods provide a more flexible and universally applicable learning paradigm for GNNs.

However, there are significant limitations in current graph contrastive learning when dealing with complex graph data, especially in capturing high-order relationships and global structures. Despite the effectiveness of graph contrastive learning in many scenarios, it primarily focuses on learning similarity in low-order or local structures, leading to neglect of rich high-order relationships and global structural information in graph data. High-order structures carry rich information and are crucial for understanding the essence of the entire graph. Traditional graph contrastive learning methods often struggle to capture such complex high-order relationships by learning simple embedding vectors for nodes or edges. Additionally, a comprehensive understanding of global structures is indispensable for capturing the overall characteristics of a graph. The global structure of a graph provides an important perspective for understanding its entirety. However, existing methods fall short in capturing and utilizing high-order relationships and global structures, which affects the depth of model understanding of complex graph structures and limits the improvement of model generalization capabilities.

To overcome these limitations, we propose a novel graph contrastive learning method, namely Tensor-Perspective Graph Contrastive Learning (TP-GCL). The core idea of TP-GCL is to transform the graph into a hypergraph through clique expansion and utilize high-order adjacency tensors to represent the hypergraph. This representation serves as a contrasting view to comprehensively capture its complex structural information. We further explore the differences and similarities between the anchor graph and the tensorized hypergraph. By constructing a tensorized hypergraph perspective at a higher level, TP-GCL enhances the understanding and modeling of relationships between nodes in the graph, accurately capturing global information and improving the model’s understanding of the overall graph structure. In contrast to traditional graph contrastive learning methods, TP-GCL avoids the information bias caused by random edge dropping and feature masking, ensuring stability and consistency in the learned graph representations. Our main contributions are as follows:

1. We introduce a novel tensor perspective-based graph contrastive learning method that comprehensively captures the complex structural information of graphs and delves into the crucial role of high-order relationships and interactions among multiple nodes in graph contrastive learning.
2. Experimental results demonstrate the significant superiority of our approach compared to baseline methods, particularly in capturing graph node features and relationships.

## 2 Related work

### 2.1 Graph neural network

GNNs, as a class of neural network models designed for graph data, have demonstrated significant potential in various fields. The fundamental idea behind GNNs is to facilitate information propagation and aggregation among nodes through their connecting relationships, thereby forming node representations. Graph Convolutional Network (GCN) (Kipf and Welling, 2016) employs convolutional operations to propagate information on the graph, updating each node’s representation by aggregating information from its neighbors. Graph Attention Network (GAT) (Veličković et al., 2017), on the other hand, introduces attention mechanisms, assigning different weights to representations of nodes with respect to their neighbors, enabling more flexible information aggregation. GraphSAGE (Hamilton et al., 2017) samples neighboring nodes and aggregates their representations, enabling the model to handle large-scale graph data. GNN-BC (Yang et al., 2022) proposes an innovative graph neural network architecture that maps node attributes and topological structures to distinct representations, introducing exclusivity to reduce redundancy between these two representations. RAW-GNN (Jin et al., 2022) presents a graph neural network framework based on random walk aggregation, utilizing breadth-first and depth-first random walks to gather homogeneous and heterogeneous information. LGLP (Cai et al., 2020) transforms the graph link prediction problem into a node classification problem in a line graph, effectively learning features of the target links. DeepMAP (Ye et al., 2020) introduces a scheme to capture complex high-order interactions around each node, extending convolutional neural networks to arbitrary graphs by generating aligned node sequences and constructing perception domains for each node. RTGNN (Zhao et al., 2022) proposes a multi-view graph representation learning method by introducing tensors to enhance relationships between inter-graph and intra-graph features. DeepGNAS (Feng et al., 2023) presents a generative process for graph neural networks, utilizing an innovative two-stage search space to automatically construct efficient and transferable deep graph neural network models in a modular manner.

### 2.2 Graph contrastive learning

Contrastive learning, notable for its ability to obtain discriminative graph representations without the need for external label supervision, has garnered significant attention, especially in fields like computer vision. As a self-supervised learning paradigm, its objective is to identify subtle similarities and differences between samples, imparting semantically rich representations to node features. Recently, in the field of graph neural networks, contrastive learning has undergone substantial evolution. DGI (Veličković et al., 2018) stands out by maximizing node-level mutual information in the graph, thereby elevating the effectiveness of graph representation learning. GMI (Peng et al., 2020) introduces the concept of graph mutual information, strengthening graph representations by maximizing mutual information between node pairs. MVGRL (Hassani and Khasahmadi, 2020), taking a multi-view perspective, enhances graph representation learning by introducing multiple graph views. GraphCL (You et al., 2020) focuses on a universal graph contrastive learning method and further extends its contributions by introducing four innovative graph enhancement techniques. GraphMAE (Hou et al., 2022) concentrates on improving graph generation through self-supervised pretraining, employing mask strategies and scaled cosine loss. H-GCL (Zhu et al., 2023), by constructing the hypergraph view of a graph, enables more comprehensive incorporation of high-order graph information into graph representations, providing richer information for graph embedding generation.

However, current GNNs and graph contrastive learning methods face a series of limitations when dealing with complex graph structures, higher-order relations, and node interactions in the real world. They often struggle to thoroughly explore the global dependencies within graph data, and their representations fall short in capturing the diversity. To address these challenges, we propose a tensor-perspective graph contrastive learning method, TP-GCL. This method aims to comprehend the inherent structure of the graph and the intricate associations between nodes more accurately and comprehensively, utilizing a tensor perspective. TP-GCL emphasizes higher-level graph representation learning by introducing higher-order relations and adjacency tensor representations. It captures the complexity of the graph more comprehensively and deeply, enhancing the model’s understanding of node relationships and improving its perception of the overall graph structure.

## 3 Definition of the problem

### 3.1 Definition 1 (tensorized hypergraph)

In tensorized hypergraphs, we contemplate tensorization of the hypergraph to more comprehensively represent its connectivity. For a given hypergraph G=(V,H) , where V is the set of nodes and H is the set of hyperedges, we describe the connectivity in the hypergraph through tensorization. Let $T\in R^{n\times n\times \ldots \times n}$ be the adjacency tensor of the hypergraph, where n is the number of nodes in the hypergraph, and the tensor elements $T\left[i_{1},i_{2},\ldots ,i_{k}\right]$ signify the presence or absence of an edge connecting nodes $v_{i_{1}},v_{i_{2}},..,v_{i_{k}}$ in the hypergraph.

### 3.2 Definition 2 (Graph contrastive learning)

Graph contrastive learning aims to achieve an effective measurement of the similarity and dissimilarity between graphs $G_{1}$ and $G_{2}$ by learning a mapping function $f\left(\cdot \right)$ . For a given pair of graphs $G_{1}=\left(V_{1},E_{1}\right)$ and $G_{2}=\left(V_{2},E_{2}\right)$ , this function ensures that the feature vectors $f\left(v_{i}\right)$ and $f\left(u_{i}\right)$ for any nodes $v_{i}\in V_{1}$ and $u_{i}\in V_{2}$ are well-represented in a low-dimensional space. Graph contrastive learning methods are predominantly based on mutual information (MI), with the objective of evaluating the degree of correlation between different variables and maximizing mutual information. To accomplish this goal, graph contrastive learning necessitates defining a contrastive loss function L to quantify the similarity between the two graphs. The key symbols used in this paper are detailed in Table 1.

**Table 1:** Main Symbols.

Symbols	Definition
G=(V,H)	Hypergraph obtained through clique expansion.
T	Adjacency tensor.
$f\left(\cdot \right)$	Mapping function in graph contrastive learning.
L	Contrastive loss function.
C	Clusters obtained after graph clustering.
k	Order of the clique.

## 4 Definition of the problem

**Figure 1:**
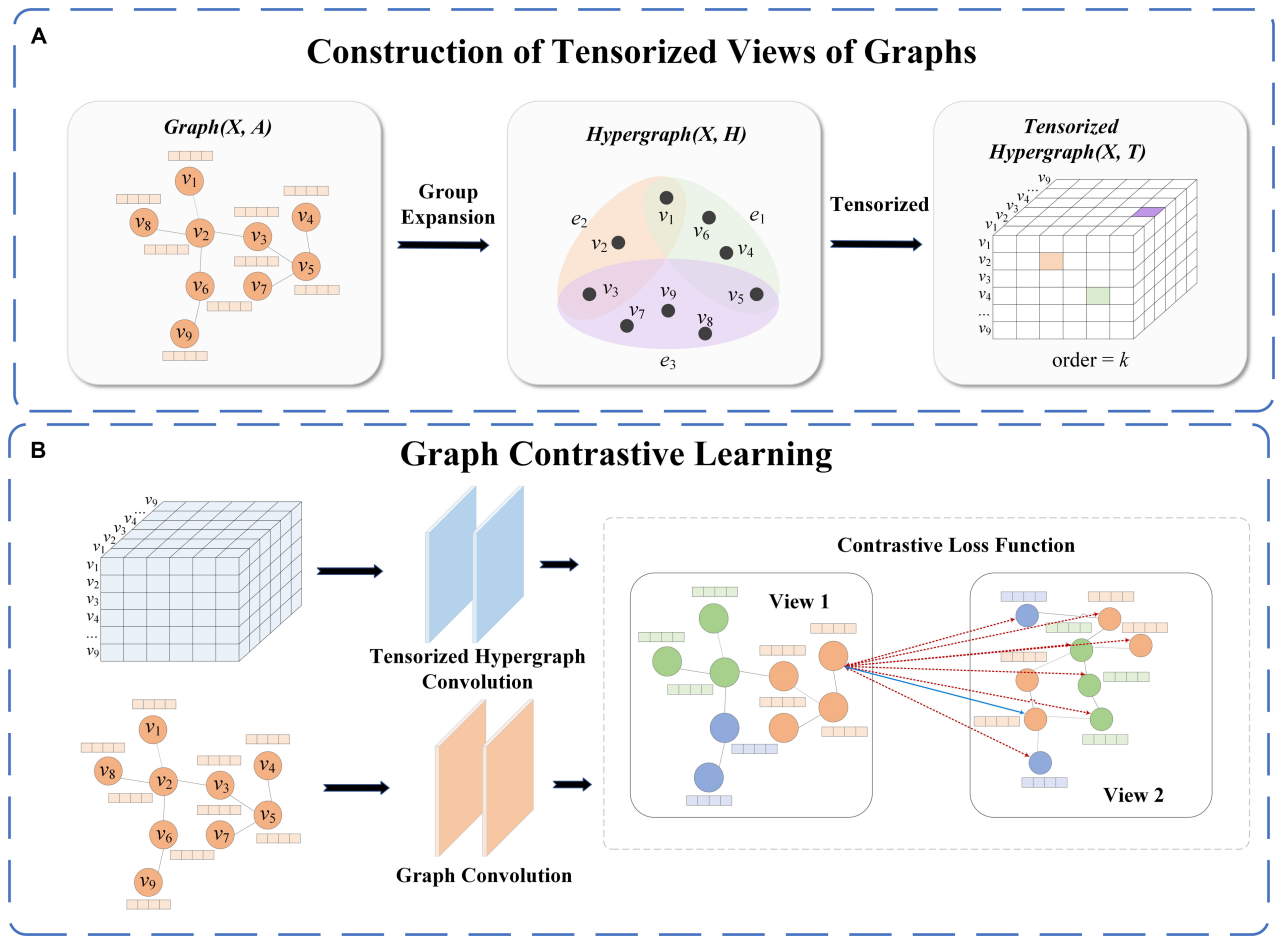
Framework diagram in this paper. [**(A)** represents the construction of the hypergraph view and **(B)** represents the model architecture diagram].

This section introduces a graph contrastive learning method based on a tensor perspective. As illustrated in Figure 1, TP-GCL consists of two main parts. The first part involves the construction of the tensorized hypergraph view module. In this process, we first utilize a clique expansion method to transform a regular graph into a hypergraph, aggregating nodes from the regular graph into high-order nodes in the hypergraph while considering the connectivity between nodes. Subsequently, we use high-order adjacency tensors to represent the tensorized hypergraph, providing a more comprehensive description of its complex structural features in tensor form. The second part is the graph contrastive learning module. The original graph serves as the anchor graph, and the tensorized hypergraph is used for comparison with the anchor graph. To evaluate the dissimilarity and similarity between the two views, positive and negative samples are designated, ensuring that similar nodes are closer in the representation space, while dissimilar nodes are farther apart.

### 4.1 Construction of tensorized views of graphs

Given the original graph G=(V,E,X) , where V is the node set, E is the edge set, $X\in R^{N\times d}$ is the node feature matrix, N is the number of nodes, and d is the feature dimension. Through the clique expansion method, we transform the graph G into a hypergraph $G^{\prime }=\left(V,E^{\prime },X\right)$ to capture higher-order relational information. Specifically, considering a node set V in graph G , we seek a subset C of highly connected nodes. A k order clique C is defined as a subset with k nodes, where k is a positive integer, formally represented as Eq. (1):


(1) $$C=\left\{v_{i_{1}},v_{i_{2}},..,v_{i_{k}}\right\},$$

where $v_{i_{j}}\in V$ , we progressively expand the k order clique C by adding nodes highly connected to the nodes in C until no new nodes can be added. This process results in a set of k order cliques $\left\{C_{1},C_{2},..,C_{m}\right\}$ , where each k order clique $C_{i}$ represents a hypernode, i.e., a set of hyperedges $E^{\prime }$ .

Through the above-mentioned method, we obtained a hypergraph $G^{\prime }$ based on the original graph. Furthermore, we construct a tensorized hypergraph to represent the higher-order structural information of $G^{\prime }$ . $G^{\prime }$ is different from ordinary graphs. Hypergraphs are described using an incidence matrix ( $H\in R^{n\times |E|}$ ), while ordinary graphs are described using an adjacency matrix ( $A\in R^{n\times n}$ ). We define the adjacency tensor $T\in R^{n\times n\times \ldots \times n}$ for the hypergraph, where the order of T is represented by m , indicating the cardinality of the hyperedge $e^{\prime }\in E^{\prime }$ . The adjacency tensor T is formulated as Eq. (2).


(2) $$T_{i_{1},i_{2},\ldots ,i_{q}=\left\{\begin{array}{c} \beta ,if\;\left\{i_{1},i_{2},\ldots ,i_{q}\right\}\;is\;connected,\\ 0,\;\;otherwise. \end{array}\right.}$$

where ***β*** represents the weight of edge $\left\{i_{1},i_{2},\ldots ,i_{q}\right\}$ , obtained by computing the weight for each permutation combination (adjacency tensor coefficients), formally represented as Eq. (3) and Eq. (4),


(3) $$\beta_{i_{1},i_{2},\ldots ,i_{q}}=\frac{c}{\alpha },$$

(4) $$\alpha =\underset{r_{1},r_{2,},\ldots ,r_{c}\geq 1,\;\;\overset{c}{\underset{i=1}{\sum }}r_{i=m}}{\sum }\left(\begin{array}{c} m\\ r_{1},r_{2,},\ldots ,r_{c} \end{array}\right),$$

where α represents the number of corresponding permutations, and α is a polynomial coefficient with additional constraints $r_{1},r_{2,},\ldots ,r_{c}\neq 0$ . This tensor construction method maximally preserves the original hyperedge structure, further reflecting the associative patterns between different nodes in the hypergraph. To better understand this process, we provide an illustrative example:

*Example 4.1.* For a given hyperedge $e_{1}^{\prime }=\left\{v_{1},v_{2},v_{3}\right\}$ , to construct a 2-order adjacency tensor, we need to consider all permutations of length **2** for the node $v_{1},v_{2},v_{3}$ . This means that hyperedge $e_{1}^{\prime }$ needs to choose **3** nodes from **2** positions for permutation, which will result in one of the **3** nodes being discarded, yielding $\left\{v_{1},v_{2}\right\},\left\{v_{1},v_{3}\right\},\left\{v_{2},v_{3}\right\}$ . Then, calculate the adjacency tensor coefficient as 23 , where the numerator is the cardinality of hyperedge $e_{1}^{\prime }$ , and the denominator is the number of permutations in this case.

### 4.2 Graph contrastive learning

Graph contrastive learning aims to extract effective node features by comparing the feature differences between the original graph and the tensorized hypergraph. To comprehensively understand the data from different perspectives, we employ GCN for encoding learning on the original graph G . This process maps node features from a high-dimensional space to a low-dimensional feature space denoted as $R^{N\times k}$ , resulting in the node feature vector $Z_{1}$ under the original graph, formulated as Eq. (5),


(5) $$Z_{1}=\sigma \left({\hat{D}}^{-\frac{1}{2}}\cdot \hat{A}\cdot {\hat{D}}^{-\frac{1}{2}}\cdot X\cdot W\right),$$

where $\hat{A}=A+I$ denotes the adjacency matrix with self-loops, ${\hat{D}}_{ij}=\sum_{j=0}{\hat{A}}_{ij}$ represents the diagonal matrix, $\sigma \left(\cdot \right)$ signifies the non-linear transformation function, and W corresponds to the learnable weight matrix. Additionally, alignment between node features and adjacency tensor is achieved through a learnable weight matrix. The outer product pooling technique is then employed on the adjacency tensor to perform tensor convolution on $G^{\prime }$ , facilitating information aggregation, formulated as Eq. (6),


(6) $$Z_{2}=\sigma \left(\hat{T}\cdot X\cdot \Theta \right),$$

where $\sigma \left(\cdot \right)$ represents the non-linear transformation function, $\hat{T}$ signifies the insertion of a self-loop matrix into the adjacency tensor, Enhancing the model’s focus on node-specific information helps in learning more comprehensive node representations, $\hat{T}=T+\overset{\varphi \left(i\right)}{\underset{j,j\neq i}{\sum }}\xi_{ij}$ , ξ is defined as **1** when i=j , otherwise it is **0**, and $Z_{2}$ denotes the tensor perspective of node representation information. To minimize the similarity between positive samples and maximize the similarity between negative samples, a contrastive loss function is employed to enhance the discriminative power of node embeddings. For embeddings of the same node in two different views, we treat the same node from different views as positive samples and consider all other nodes as negative samples. Furthermore, we optimize the positive sample pairs $\left(z_{1,i},z_{2,i}\right)$ in a pairwise manner, formulated as Eq. (7),


(7) $$L_{\left(z_{1,i},z_{2,i}\right)}=\log\frac{e^{\theta \left(z_{1,i},z_{2,i}\right)/\tau }}{e^{\theta \left(z_{1,i},z_{2,i}\right)/\tau }+\sum_{k=1}^{N}1_{\left[k\neq i\right]}e^{\theta \left(z_{1,i},z_{2,i}\right)/\tau }},$$

where τ is a temperature parameter used to measure and adjust the distribution of similarities between samples in $L_{\left(z_{1,i},z_{2,i}\right)}$ . $1_{\left[k\neq i\right]}\in \left\{0,1\right\}$ is an indicator function, taking the value **1** only when k=i . Considering the symmetry between views, we employ a symmetry loss function to reflect the symmetric features of node embeddings between the two views. Ultimately, our loss function $L_{\left(z_{1,i},z_{2,i}\right)}$ is formulated as Eq. (8),


(8) $$L=\frac{1}{2N}\overset{N}{\underset{i=1}{\sum }}\left(L\left(z_{1,i},z_{2,i}\right)+L\left(z_{2,i},z_{1,i}\right)\right),$$

## 5 Experiments

In this section, we first introduce the datasets utilized in our experiments. Subsequently, we compare our method with baseline approaches and conduct relevant ablation experiments. Finally, we perform additional experiments to further validate the superiority of the proposed method presented in this paper.

### 5.1 Datasets

To validate the effectiveness of TP-GCL, we designed two sets of experiments, namely node classification tasks and graph classification tasks. The details of the datasets are provided in Table 2.

**Table 2:** Statistics of datasets used in experiments.

Dataset	Nodes	Edges	Features	Classes
Cora	2,708	5,278	1,433	7
Citeseer	3,327	4,552	3,703	6
PubMed	19,717	44,324	500	3

Our aim is to comprehensively evaluate the performance of the TP-GCL model in node classification. Node classification tasks focus on categorizing nodes with different features and labels. The datasets Cora, Citeseer, and PubMed belong to the academic network domain, where nodes represent papers, and edges represent citation relationships between papers. By utilizing these datasets, we validate the effectiveness and generalization capability of the TP-GCL method on graph data of various sizes and scales.

### 5.2 Baselines

The baseline models for node classification tasks can be categorized into two groups. The first group includes semi-supervised learning methods such as ChebNet (Tang et al., 2019), GCN (Kipf and Welling, 2016), GAT (Veličković et al., 2017), GraphSAGE (Hamilton et al., 2017), which utilize node labels during the learning process. The second group comprises self-supervised methods, including DGI (Veličković et al., 2018), GMI (Peng et al., 2020), MVGRL (Hassani and Khasahmadi, 2020), GraphCL (You et al., 2020), GraphMAE (Hou et al., 2022), H-GCL (Zhu et al., 2023), United States-GCL (Zhao et al., 2023) which do not rely on node labels. The proposed TP-GCL in this paper also falls into the category of self-supervised graph contrastive learning methods.

### 5.3 Experiment implementation details

During the experimental process, we utilized the NVIDIA A40 GPU, equipped with 48GB of VRAM and 80GB of CPU memory. The deployment of TP-GCL was supported by PyTorch 1.12.1, PyTorch Geometric, and the PyGCL library. The code for our experiments will be made publicly available in upcoming work. For specific optimal parameter settings, please refer to Table 3. As shown in the table, Training epochs indicates the total number of epochs required for training, Learning rate controls the step size of model parameter updates, Weight decay is a regularization coefficient used to prevent overfitting, τ is the temperature coefficient used to set the focus on hard negative samples during contrastive learning, and Hidden dimension determines the size of the hidden layer, affecting the complexity and expressive power of feature learning.

**Table 3:** Detailed parameter setting.

Dataset	Training epochs	Learning rate	Weight decay	𝜏	GCN layers	TGCN layers	Hidden dimension
Cora	200	0.005	0	0.7	2	1	512
Citeseer	200	0.01	0	0.7	2	2	512
PubMed	400	0.005	5e-4	0.6	2	2	256

### 5.4 Experimental results

We validated the effectiveness of TP-GCL on node classification tasks, and Table 4 presents the performance comparison on the Cora, Citeseer, and Pubmed datasets.

**Table 4:** The performance of accuracy on node classification tasks.

Model	Use data	Cora	Citeseer	PubMed
ChebNet	X, A, Y	81.2 ± 0.6	69.8 ± 0.5	74.4 ± 0.4
GCN	X, A, Y	81.5 ± 0.7	70.3 ± 0.7	79.0 ± 0.3
GAT	X, A, Y	83.0 ± 0.7	72.5 ± 0.7	79.0 ± 0.3
GraphSAGE	X, A, Y	77.2 ± 0.3	67.8 ± 0.3	77.3 ± 0.5
DGI	X, A	81.7 ± 0.6	71.5 ± 0.7	77.3 ± 0.6
GMI	X, A	82.7 ± 0.2	73.0 ± 0.3	80.1 ± 0.2
MVGRL	X, A	82.9 ± 0.7	72.6 ± 0.7	79.4 ± 0.3
GraphCL	X, A	82.5 ± 0.2	72.8 ± 0.3	77.5 ± 0.2
GraphMAE	X, A	84.2 ± 0.4	73.4 ± 0.4	81.1 ± 0.4
H-GCL	X, A	84.8 ± 0.5	74.2 ± 0.3	83.2 ± 0.6
USA-GCL	X, A	**85.9 ± 0.4**	**75.9 ± 0.6**	82.3 ± 0.6
TP-GCL	X, A	**85.4 ± 0.6**	**74.5 ± 1.8**	**83.8 ± 0.3**

Here, X represents node features, Y represents labels, and A represents the adjacency matrix.

The results in Table 4 clearly demonstrate the superior performance of TP-GCL in node classification tasks. TP-GCL exhibits high accuracy on three different datasets, Cora, Citeseer, and PubMed, surpassing other baseline models. This can be attributed to several advantages:

1. TP-GCL comprehensively captures the structural features of graphs in complex spaces using high-order adjacency tensors. Compared to traditional methods, high-order tensor representations provide richer information, facilitating a better understanding of both local and global structures in the graph. This allows TP-GCL to more accurately learn abstract representations of nodes.
2. Through the contrastive learning mechanism of anchor graph-tensorized hypergraphs, TP-GCL sensitively learns subtle differences and similarities between nodes. This learning approach makes TP-GCL more discriminative, enabling accurate differentiation of nodes from different categories.

### 5.5 Hyperparametric sensitivity

**Figure 2:**
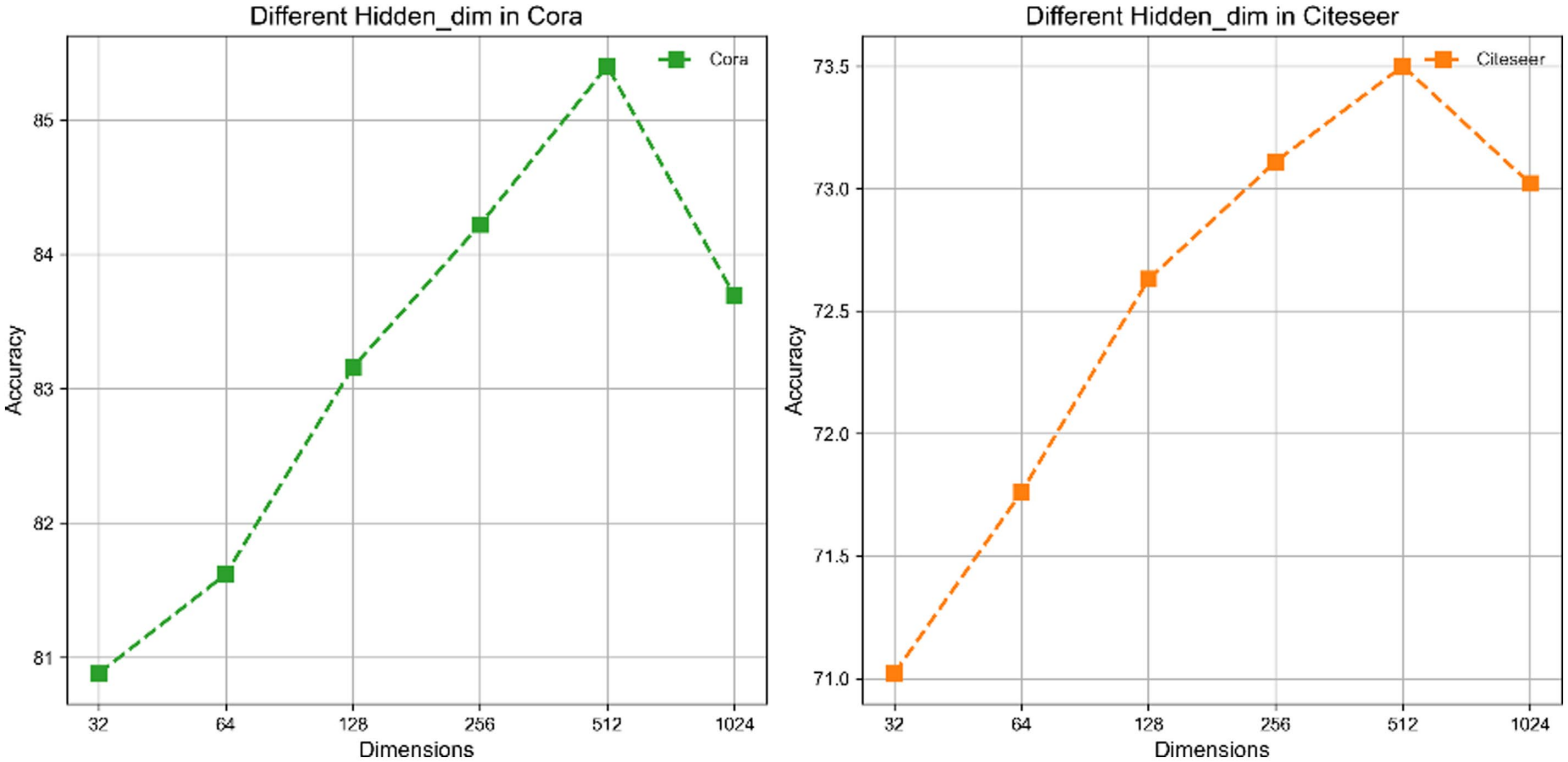
Performance of hidden layer dimension on Cora and Citeseer.

Our research focuses on an in-depth analysis of key hyperparameters such as hidden layer dimension, Tau value, and learning rate. Firstly, the hidden layer dimension plays a crucial role in the performance of TP-GCL. By adjusting the dimension of the hidden layer, we explored the impact of different dimensions on the model’s performance on the Cora and Citeseer datasets. As shown in Figure 2, the results indicate that increasing the dimension of the hidden layer within the range of [32 ~ 512] enhances the fitting capability of TP-GCL, with the optimal performance reached when the dimension equals 512. This is because a higher-dimensional hidden layer helps capture more complex data patterns. However, excessively high dimensions, such as 1,024, can lead to overfitting.

**Figure 3:**
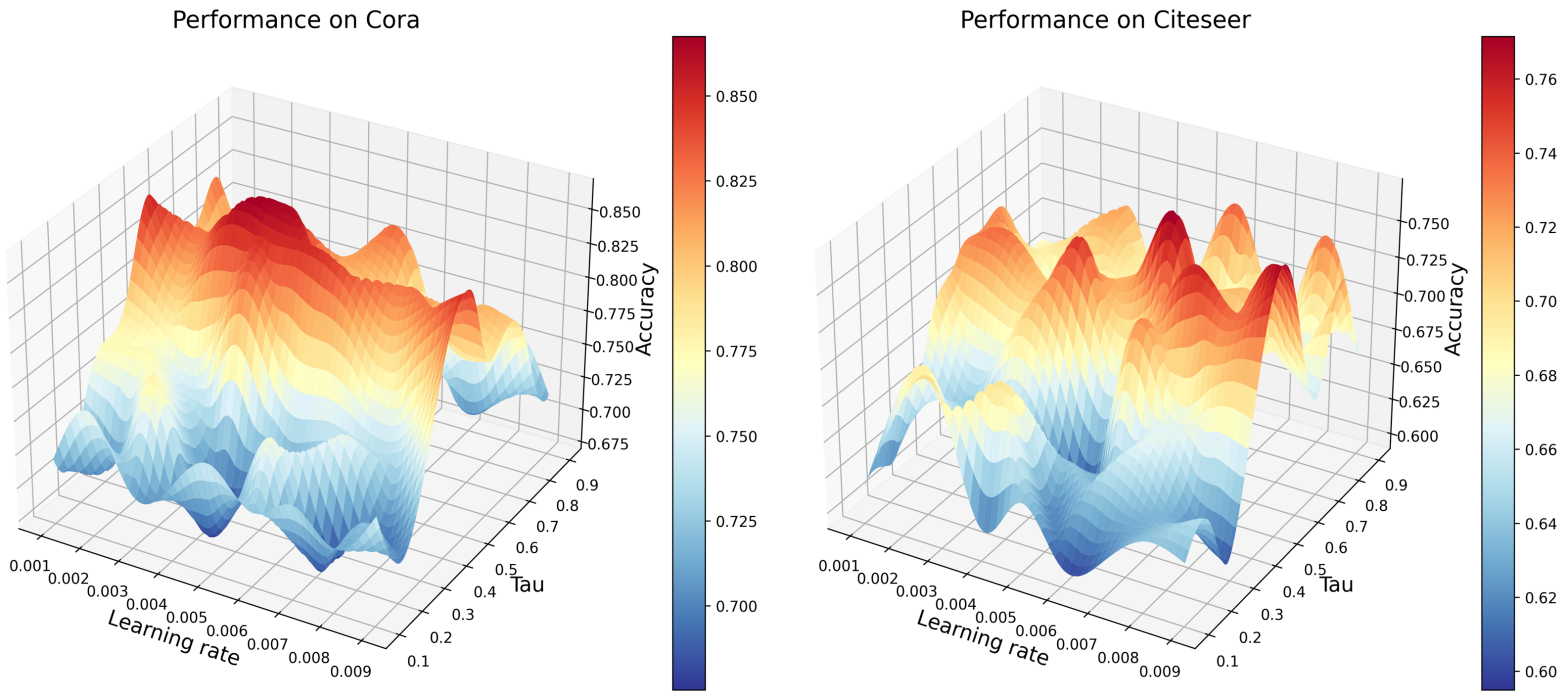
Performance of different learning rates and tau values on the Cora and Citeseer datasets.

Next, we focused on the hyperparameters Tau and learning rate. Tau is typically used to control the smoothness of the distribution of similarities in contrastive learning, while the learning rate is used to regulate the speed of model parameter updates during training. We plotted the parameter space with the x-axis representing the learning rate in the range [0.001 ~ 0.009], the y-axis representing Tau in the range [0.1 ~ 0.9], and the z-axis representing the accuracy of node classification, as shown in Figure 3.

From Figure 3, it can be observed that the variation in accuracy is influenced by changes in the learning rate under different Tau values. When Tau values are low (0.1–0.3), combinations within the learning rate range of 0.001–0.004 generally result in lower accuracy. This might be attributed to the slower parameter update speed caused by the lower learning rates in this range, preventing the model from fully utilizing information in the dataset and thereby hindering accurate node differentiation. Additionally, lower Tau values imply more sensitivity in similarity calculations, potentially causing similarity to concentrate too much between nodes, making effective node discrimination challenging and consequently reducing accuracy. On the other hand, when Tau values are high (0.6–0.9), combinations within the learning rate range of 0.008–0.009 exhibit relatively higher accuracy. This is possibly due to the higher learning rates in this range accelerating the model’s parameter update speed, aiding the model in better learning the dataset’s features. Furthermore, higher Tau values smooth out the similarity distribution, reducing the model’s sensitivity to noise and subtle differences in the data, allowing the model to better discriminate between nodes and thereby improving accuracy.

## 6 Conclusion

In response to the challenges posed by existing graph neural network methods in capturing global dependencies and diverse representations, as well as the difficulty in fully revealing the inherent complexity of graph data, this paper proposes a novel tensor-perspective graph contrastive learning method, TP-GCL. The aim is to comprehensively and deeply understand the structure of graphs and the relationships between nodes. Firstly, TP-GCL transforms graphs into tensorized hypergraphs, introducing higher-order information representation while preserving the original topological structure of the graph. This addresses the limitations of existing methods in capturing the complex structure of graphs and relationships between nodes. Subsequently, in TP-GCL, we delve into the differences and similarities between anchor graphs and tensorized hypergraphs to enhance the model’s sensitivity to global information in the graph. Experimental results on public datasets demonstrate a comprehensive evaluation of TP-GCL’s performance, validating its outstanding performance in the analysis of complex graph structures.

## Data availability statement

The original contributions presented in the study are included in the article/supplementary material, further inquiries can be directed to the corresponding author/s.

## Author contributions

ML: Formal analysis, Investigation, Methodology, Validation, Visualization, Writing – original draft, Writing – review & editing. LM: Formal analysis, Methodology, Writing – original draft, Writing – review & editing. ZY: Conceptualization, Supervision, Validation, Writing – review & editing. YY: Formal analysis, Methodology, Software, Writing – review & editing. SC: Formal analysis, Methodology, Writing – review & editing. YX: Data curation, Validation, Writing – review & editing. HZ: Conceptualization, Funding acquisition, Supervision, Writing – review & editing.
